# Automated Integration of Trees and Traits: A Case Study Using Paired Fin Loss Across Teleost Fishes

**DOI:** 10.1093/sysbio/syx098

**Published:** 2018-01-09

**Authors:** Laura M Jackson, Pasan C Fernando, Josh S Hanscom, James P Balhoff, Paula M Mabee

**Affiliations:** 1 Department of Biology, University of South Dakota, 414 East Clark St., Vermillion, SD 57069, USA; 2 Renaissance Computing Institute, University of North Carolina, 100 Europa Drive Suite 540, Chapel Hill, NC 27517, USA

**Keywords:** Ancestral state reconstruction, bioinformatics pipeline, evolutionary morphology, fishes, inference, missing data, ontology, paired fins, phenotype

## Abstract

Data synthesis required for large-scale macroevolutionary studies is challenging with the current tools available for integration. Using a classic question regarding the frequency of paired fin loss in teleost fishes as a case study, we sought to create automated methods to facilitate the integration of broad-scale trait data with a sizable species-level phylogeny. Similar to the evolutionary pattern previously described for limbs, pelvic and pectoral fin reduction and loss are thought to have occurred independently multiple times in the evolution of fishes. We developed a bioinformatics pipeline to identify the presence and absence of pectoral and pelvic fins of 12,582 species. To do this, we integrated a synthetic morphological supermatrix of phenotypic data for the pectoral and pelvic fins for teleost fishes from the Phenoscape Knowledgebase (two presence/absence characters for 3047 taxa) with a species-level tree for teleost fishes from the Open Tree of Life project (38,419 species). The integration method detailed herein harnessed a new combined approach by utilizing data based on ontological inference, as well as phylogenetic propagation, to reduce overall data loss. Using inference enabled by ontology-based annotations, missing data were reduced from 98.0% to 85.9%, and further reduced to 34.8% by phylogenetic data propagation. These methods allowed us to extend the data to an additional 11,293 species for a total of 12,582 species with trait data. The pectoral fin appears to have been independently lost in a minimum of 19 lineages and the pelvic fin in 48. Though interpretation is limited by lack of phylogenetic resolution at the species level, it appears that following loss, both pectoral and pelvic fins were regained several (3) to many (14) times respectively. Focused investigation into putative regains of the pectoral fin, all within one clade (Anguilliformes), showed that the pectoral fin was regained at least twice following loss. Overall, this study points to specific teleost clades where strategic phylogenetic resolution and genetic investigation will be necessary to understand the pattern and frequency of pectoral fin reversals.

How often—across all 33,000$+$ species of teleost fishes—were pectoral and pelvic fins lost? Are they ever regained? The information required to answer these straightforward questions regarding character evolution, namely trait data and a relatively resolved species-level phylogeny, has not been readily available at the scale of tens of thousands of species until recently (Dececchi et al. 2015; Hinchliff et al. 2015). Investigators interested in addressing such macroevolutionary questions have been confronted with challenges when attempting to combine trait data with phylogenies at magnitudes not readily supported by current tools (Harris and Arbuckle 2016; Hunt and Slater 2016). Alfaro et al. (2009) suggest that some of these challenges involve producing and manipulating large-scale phylogenies. In addition, the development of larger phylogenies has further exposed the lack of resources necessary to visualize and interpret results (Harmon et al. 2013). Not only are tools for mapping large trait data sets on phylogenies lacking, but phylogenetic programs also lack the scalability necessary to approach macroevolutionary questions (Harmon etal. 2013; Harris and Arbuckle 2016; Hunt and Slater 2016). Although methods to investigate trait evolution at larger scales are improving (e.g., iTOL: Letunic and Bork 2007; Harmon et al. 2013), many still lack the ability to integrate data sets across multiple data formats and analyses.

The peculiar absence of paired fins in fishes such as eels (Nelson 2006) has fascinated scientists since at least the time of Aristotle (Ogle 1882; Leunissen 2010). Notably, over the past 40 years, ichthyologist Nelson (1976, 1984, 1994, 2006) documented the absence of pelvic fins in members of 92 teleost families (Nelson 1990), which represent nearly one-quarter of all fish families. This led him to conclude that pelvic fins have been independently lost at least 50 times (Nelson 1990). His analysis, as well as subsequent ones (Yamanoue et al. 2010); however, did not consider phylogenetic relationships, in which absence or presence at an ancestral node determines the number of putative losses. Further, Nelson (2006) summarized data at the family level, for example, for Ophichthidae: “pectoral fins present or absent,” often without naming the species associated with a particular condition or citing the primary literature in this regard. In contrast to data on pelvic fins, the frequency of pectoral fin loss is poorly documented, with few exceptions (Britz 2010; Yamanoue et al. 2010; Brown et al. 2011). Further, whether pectoral or pelvic fins may have been regained following loss remains uncertain (Nelson 1990; Yamanoue et al. 2010). Though there are known exceptions to Dollo’s law, that is, that the loss of a complex trait is evolutionarily irreversible (Gould 1970; Farris 1977), questions concerning the taxonomic scope, frequency of reversal, and potential genetic bases (Collin and Miglietta 2008) remain unanswered.

Addressing any broad-scale question concerning the evolution of traits requires a comprehensive source of data. Such large data sets must be readily extractable and computable, as manual aggregation from a dispersed literature is essentially intractable. Free text phenotypic descriptions from the literature that are tagged with appropriate ontology terms [via Uniform Resource Identifiers (URIs)] provide semantic information that allows for automated collection and computation of morphological data across species (Deans et al. 2015; Dececchi et al. 2015). For paired fin data, we used the Phenoscape Knowledgebase (KB; kb.phenoscape.org) as the source for computable phenotypes across vertebrates. The KB contains ontology-annotated phenotypic data based primarily on published character matrices (Dececchi et al. 2015), but also some monographic treatments (e.g., Nelson 2006; Dececchi et al. 2016). It is particularly enriched in vertebrate skeletal features, such as fins, limbs, and their support structures. The ontology-based data uniquely allow inference of the presence or absence of a phenotypic feature based on indirect descriptions of the feature or its parts. For example, such inference has been shown to greatly enlarge the available data (Dececchi et al. 2015), a desirable feature here given the paucity of direct statements by authors concerning the presence/absence of paired fins. Thus, rather than combing through publications relevant to 33,000$+$ teleost species and manually compiling a matrix encompassing the full scope of data relevant to our question, ontology-annotated data, including both author-asserted and machine reasoned data populating the KB, can be automatically exported into a data set for analysis.

Obtaining a fully resolved phylogenetic tree, in this case for all extant and extinct teleost species, is a major challenge for any large-scale analysis of trait evolution. For teleost fishes, the recent literature includes some well-resolved and broad-scale trees based on molecular data (Near et al. 2012; Betancur et al. 2013), though there are several impediments to their use. First, they involve only a subset of teleost species; for example, Betancur et al. (2013) sampled 1410 species. Second, when the terminal taxa are at a supra-specific level, for example, families, orders, and superorders as in the Near et al. (2012) tree, it is difficult to know which species were included in these groupings at the time of analysis (though actual species sampled were provided in the case of Near et al. 2012). Further, even if authors report the species included in an analysis (as in Near et al. 2012), the effort to add these manually to a large tree is untenable. Third, assembling published trees that likely differ in topology becomes increasingly difficult to accomplish manually at large scales, and requires automated methods of tree synthesis. Thus we accessed the Open Tree of Life project (Open Tree; http://opentreeoflife.org) for a comprehensive tree for teleosts at the species level (Hinchliff et al. 2015). The Open Tree dynamically constructs a tree by synthesizing published phylogenies along with taxonomic data using the “propinquity” supertree pipeline (Redelings and Holder 2017). The output includes detailed provenance reports (e.g., node support, conflicts, and resolutions) associated with nodes resolved by a source other than the reference taxonomy.

While comprehensively synthesizing all available paired fin trait data for teleosts was the first step in investigating patterns in fin evolution, we also applied new methods to extend existing data, while minimizing overall data loss. This was achieved by several means. First, we used inferred trait data (Dececchi et al. 2016) to fully utilize existing information on paired fins in the literature. For example, if an author reports that part of a fin is present in a particular taxon, then the fin itself is inferred to be present. Second, we propagated data that investigators associated with families and genera in their matrices and descriptions to the species level, thus extending the data as intended by the authors and generating a more comprehensive matrix for ancestral state reconstruction. Finally, we improved the method of taxonomic reconciliation between taxa to which trait data are attached and taxa included in the phylogeny. This was necessary because different sources of names are used in taxonomies referenced by the Phenoscape KB and the Open Tree of Life. The total expansion of phenotypic data through ontology-based inference and taxonomically-based propagation was substantial and is a valuable model to be followed for macroevolutionary studies.

Using questions concerning the frequency of paired fin loss in fishes as an example, we demonstrate the use of new knowledge resources to address basic questions involving large-scale phenotypic evolution. Increasing the taxonomic scope makes apparent the value of these new resources, as well as deficiencies in existing methods to integrate the data. The bioinformatics pipeline developed in the process of this work reflects a set of essential requirements for large-scale macroevolutionary syntheses.

## Methods

### Large-scale Computable Phenotypic Data

To compile large-scale morphological data on pectoral and pelvic fin presence/absence across all teleost fishes we needed a publically available source containing computable phenotypic data that allowed extraction of a single matrix across a specified taxon. The Phenoscape Knowledgebase (KB) contains 21,569 character states annotated with 526,221 phenotypes for 5208 extant and fossil vertebrates from 171 comparative studies (as of October 21, 2016). Twenty-two of the 171 studies in the KB were added to fully represent the distribution of pectoral and pelvic fins across teleosts (Supplementary Table S1 available on Dryad at http://dx.doi.org/10.5061/dryad.v0s27). These studies are primarily phylogenetic, but also include monographic treatments (Dececchi et al. 2016) and reviews (Wiley and Johnson 2010). Phenotypic data from the KB are annotated with taxonomic names from the Vertebrate Taxonomy Ontology (VTO, http://purl.obolibrary.org/obo/vto/2016-1017/vto.owl; Midford et al. 2013). The VTO is built upon the National Center for Biotechnology Information (NCBI) taxonomy, which provides the hierarchical backbone for extant taxa; valid species are drawn from the expert source, the Catalog of Fishes (CoF; Eschmeyer et al. 2013). PaleoDB (Uhen et al. 2013) supplements extinct taxa (Midford et al. 2013). The VTO (July 2012) contains fewer teleost species (31,726) than the current CoF (33,191; November 20, 2017) because it has not been updated with a new version of the CoF.

**Figure 1.**
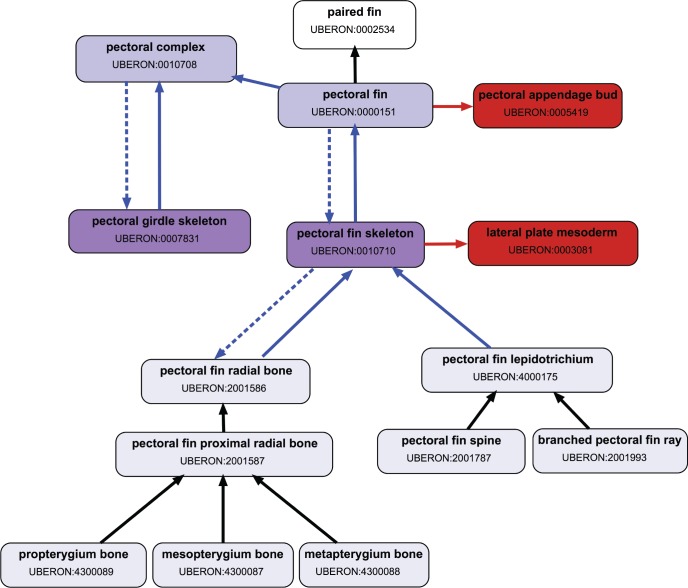
Subgraph from the Uberon anatomy ontology showing the relationships of terms associated with pectoral fin and girdle. Arrows represent logical relationships: *part_of* (blue solid), *has_part* (blue dashed), *develops_from* (red), *is_a* (black).

Characters were annotated using the Entity–Quality (EQ) formalism (Mungall et al. 2007, 2010) with Phenex software (Balhoff et al. 2010, 2014). Specifically, ontological terms and relationships representing anatomical aspects of the paired fins, girdles, their parts and developmental precursors (Fig. 1) were drawn from the Uberon multispecies anatomy ontology (http://purl.obolibrary.org/obo/uberon/releases/2016-09-07/uberon.owl; Mungall et al. 2012; Haendel et al. 2014). Quality terms that represent the variation in these anatomical entities, such as presence/absence, size, or shape, were drawn from the Phenotype and Trait Ontology (PATO, http://purl.obolibrary.org/obo/pato/releases/2016-09-15/pato.owl; Gkoutos et al. 2005). New terms were added to Uberon as driven by the curated literature, for example, the structure “pelvic intercleithral cartilage” was used in the description of the pelvic fin for gobiiform fishes (Wiley and Johnson 2010), and thus it was added to Uberon with label “pelvic intercleithral cartilage” and identifier UBERON:4300151, and related to other terms as a type of “cartilage element” (UBERON:0007844) and a type of “pelvic region element” (UBERON:0005179). Terms and relationships in Uberon were edited using the ontology editing software Protégé v4.3 (Noy et al. 2003).

Authors describe anatomical features for higher-level taxa in addition to species (e.g., Nelson 1990; Chapleau 1993; Johnson and Patterson 1993; Wiley and Johnson 2010), and we directly annotated phenotypes to the family, generic, or species level as specified by an author. For example, if an author states that a genus lacks a fin, we annotated absence (“0”) to the genus. If an author indicates that a fin is absent at the family level, we annotated absence to the family. In cases where authors describe features for supra-familial-level taxa, but do not specify family membership in the grouping, the annotation was applied to the supra-familial taxon named in the publication. For example, “sixteen principal branched caudal fin rays” is stated to characterize Osteoglossomorpha (Wiley and Johnson 2010), and thus we annotated “Osteoglossomorpha” with this character; lower taxonomic levels were not annotated. However, where authors indicate family membership, we applied annotations to the contained families. For example, synapomorphies for Lampridiformes (also “Lampriformes”) were applied to seven families, as per the authors’ description that “Lampridiformes comprise seven monophyletic families, Veliferidae, Lamprididae (also ‘Lampridae’), Stylephoridae, Lophotidae, Radiicephalidae, Trachipteridae, and Regalecidae,” (Wiley and Johnson 2010). Authors may also describe characters for higher-level taxa not contained in the VTO (e.g., “Holacanthopterygii” in Wiley and Johnson 2010) and for which member taxa were not specified; in these cases annotations were not made. Finally, in cases where the taxonomy used in a publication differed from the VTO, we annotated the anatomical features to the taxa as intended by the author. For example, some taxa included in Osmeridae by Wiley and Johnson (2010) are elevated to their own family (Plecoglossidae) in the VTO; we ensured that the characters were applied to the relevant taxa within the family Plecoglossidae.

In cases where a higher-level taxon was described as polymorphic for the presence and absence of a particular fin, we investigated the literature to determine which of its species possess or lack the fin, and we curated the species-level data from these studies into the KB. However, in cases in which a polymorphism described for a higher-level taxon could not be traced in the literature to particular species, we excluded the data for the higher-level taxon, as was the case for pelvic fin absence asserted for the catfish families Schilbidae and Siluridae (Nelson 2006).

An additional challenge for annotation of data in review papers, particularly at the family level or above, is the frequent lack of alternative character state descriptions. For example, eels (Anguilliformes) were asserted to have the apomorphic feature “pelvic girdle and fins absent” (Wiley and Johnson 2010), and although by implication one might assume that the alternative state “pelvic girdle and fins present” might apply to other fishes, it is not stated by the authors. In these cases, we annotated only the asserted state and never an implied alternative state so as to not misrepresent author intent. The guidelines developed for annotation that reflect the above conditions were added to the Phenoscape Guide to Character Annotation (http://phenoscape.org/wiki/Guide_to_Character_Annotation; Dahdul et al. 2010), and followed consistently.

### Synthetic Morphological Supermatrix

**Figure 2.**
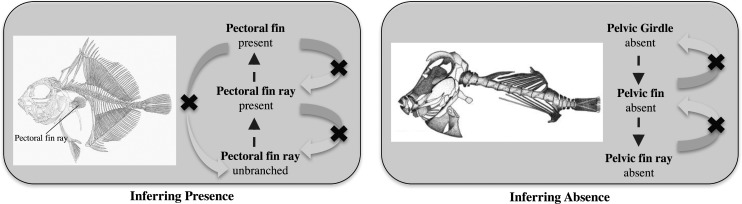
Ontology-based inference of presence and absence. Left: The presence of a structure (pectoral fin) is inferred from a quality (unbranched) of its part (pectoral fin ray), as seen in *Neocyttus rhomboidalis* (Tyler et al. 2003). Right: The absence of a pelvic girdle implies the absence of a pelvic fin and thus of a pelvic fin ray, as seen in *Acanthostracion quadricornis* (Tyler 1980). The arrows represent the direction of ontological inference, and the X’s represent relationships that are not inferred through ontological reasoning.

We used the OntoTrace tool (Dececchi et al. 2015) to retrieve a synthetic morphological supermatrix of presence/absence characters from the KB pertaining to two characters [pectoral fin: (1) present, (0) absent; pelvic fin: (1) present, (0) absent] for all teleost taxa (Supplementary Material Matrix S1 available on Dryad, 06/08/2017). The following query was used to obtain the matrix: taxon “Teleostei” and entities “pectoral fin” or “pelvic fin.” The matrix is in NeXML format (Vos et al. 2012) and contained data provenance in the metadata. OntoTrace uses inference enabled by the logical relationships among ontology-annotated data (Fig. 1) to infer the presence or absence of entities that were not directly asserted by an author. For example, the character “pectoral fin rays are unbranched” in *Neocyttus rhomboidalis* was described by Tyler et al. (2003), based on direct evidence from a voucher specimen at the American Museum of Natural History (AMNH 91746; Tyler 1980; Tyler et al. 2003). The character was annotated as Entity: “pectoral fin ray,” Quality: “branched” and the computer then inferred that the pectoral fin rays and the pectoral fin are present (Fig. 2). The converse, however, is not true; the presence of pectoral fin does not imply that any particular part is present. The absence of a paired fin, however, would be inferred from the absence of its girdle, as seen in *Acanthostracion quadricornis* (Fig. 2) based on multiple voucher specimens from the Academy of Natural Sciences of Philadelphia (e.g., ANSP 98614, ANSP 98615, ANSP 9816; Santini and Tyler 2003). This reflects domain knowledge that paired fins are never present without their supporting girdle structures. However, the opposite condition, that is, presence of a girdle, does not imply that the fin is present. There are several examples in fishes where the pectoral girdle is present, but the pectoral fin is absent (Nelson 2006), such as the black pomfret (*Parastromateus niger*), the Parona leatherjacket (*Parnoa signata*), and the fanfin (*Robia legula*). Finally, the absence of a paired fin can also be inferred from the absence of the larval fin or fin bud, but not the converse, that is, the presence of a larval fin or fin bud does not imply the presence of a fin. For example, in the William’s tonguefish (*Symphurus williamsi*; Aceves-Medina et al. 1999), the larval pectoral fin does not persist in development and thus adults lack the pectoral fin.

### Teleostei Species-level Tree

The Open Tree of Life (Hinchliff et al. 2015), which uses the “propinquity” supertree pipeline to generate synthetic trees from multiple input phylogenies and a reference taxonomy (Redelings and Holder 2017) into a single rooted synthetic supertree, which can be customized according to user preferences. The input phylogenies for Open Tree are published trees that are manually curated to align tips with Open Tree taxonomy (Rees and Cranston 2017). The propinquity supertree pipeline integrates and summarizes input phylogenies and the reference taxonomy into a single rooted synthetic supertree, which can be customized according to user preferences. Within the publicly available supertree (Open Tree 2.10; see Hinchliff et al. 2015), fourteen families had species in which the pectoral and/or pelvic fin were absent but for whom species relationships were unresolved. To provide better resolution within these families (listed below), we curated available phylogenies for them to a Teleostei tree collection (https://tree.opentreeoflife.org/curator/collections/laurajackson/teleostei) and obtained a customized (Redelings and Holder 2017) and synthetic tree (10/18/2016) for Teleostei (Supplementary Materials File S1 available on Dryad), which included the curated phylogenies. The customized synthetic tree was run excluding subspecies names and including *incertae sedis* taxa. The families targeted for phylogeny curation are those in Anguilliformes ([Anguillidae, Congridae, Cyematidae, Derichthyidae, Ophichthidae, Nettastomatidae]: Santini et al. 2013; Chlopsidae: Tang and Fielitz 2013), Percomorpha ([Chaudhuriidae, Indostomidae, Mastacembelidae, Synbranchidae]: Kawahara et al. 2008), Gymnotiformes ([Apteronotidae, Sternopygidae]: Albert 2001) and Perciformes (Trichuridae: Johnson 1986). These phylogenies are queued for inclusion in the next public version of the synthetic tree from Open Tree. All files associated with the supertree pipeline are available in Supplementary Materials File S2 available on Dryad.

### Bioinformatics Pipeline to Merge Synthetic Morphological Supermatrix and Teleostei Species-level Tree

**Figure 3.**
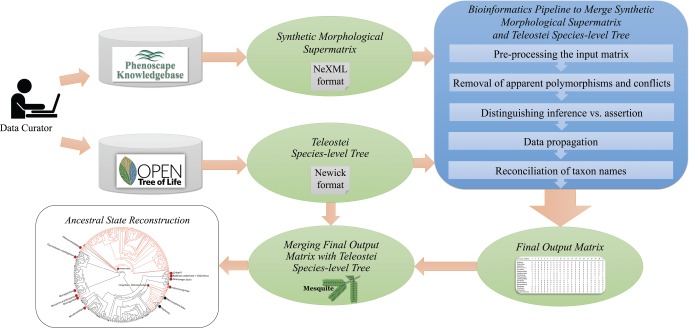
The general workflow for integrating the synthetic morphological supermatrix retrieved from the Phenoscape KB with the Teleostei species-level tree obtained from Open Tree to be used for ancestral state reconstruction.

Because of differences in taxon coverage and source taxonomies between the taxonomy used in Open Tree and the VTO taxonomy used in the supermatrix, the synthetic morphological supermatrix (Supplementary Material Matrix S1 available on Dryad) from the KB required transformation to a version that could be mapped to the Open Tree phylogeny. This was achieved by developing a bioinformatics pipeline (Fig. 3; source code available at https://doi.org/10.5281/zenodo.804488).

#### Pre-processing the input matrix.

The input to the bioinformatics pipeline was the synthetic morphological supermatrix in NeXML format obtained from OntoTrace (Supplementary Material Matrix S1 available on Dryad), which contained data for pectoral and pelvic fins. This generates a pre-processed data matrix (Supplementary Material Matrix S2 available on Dryad), which was used for the following steps (Fig. 3).

#### Removal of apparent polymorphisms and conflicts.

The matrix produced by OntoTrace enables isolation of cells with both presence and absence (represented by “0&1”) and detailed provenance reports for all cells. When a taxon is shown to have both presence and absence for one of the paired fins, it indicates a polymorphic condition, an apparent polymorphism, or a conflict in the data. Actual polymorphisms are those described at the species level and reference the same source publication. Apparent polymorphisms are described at higher taxonomic levels (e.g., genus, family) and the specific taxa that show presence and/or absence were not described by the author. Because apparent polymorphisms are not traceable to particular species and do not factor into ancestral state reconstruction, they were replaced with “?” in the matrix. Where polymorphisms were the result of conflicts in the data at the species level, they were also coded with “?” in the matrix.

#### Distinguishing inference versus assertion.

The states of “present” (1) or “absent” (0) for the pectoral or pelvic fin in the matrix may result from direct assertion, inference, or both. These were distinguished by applying an algorithm that used the associated metadata within the synthetic morphological supermatrix (Supplementary Material Matrix S1 available on Dryad) to identify whether a character state comes from an author assertion, or inference. The pre-processed data matrix (Supplementary Material Matrix S2 available on Dryad) was thus modified to distinguish between these states (Fig. 3). Cells for taxa with character states based on a direct author assertion were coded as “1” for asserted presence or “0” for asserted absence. These cells were considered to be asserted even if the same character state resulted from inference. Only cells that did not contain an author-supported assertion were counted as inferred and represented as a “2” in the matrix. Because there were no instances of only inferred absence, it was not necessary to create an alternative state for this in the matrix.

#### Data propagation.

The pre-processed data matrix (Supplementary Material Matrix S2 available on Dryad) included a substantial number of character states above the species level. These data, if used as ancestral states, could potentially enable a more accurate assessment of position and frequency of character state change. Data at higher-level internal nodes, however, are not utilized by current tools for ancestral state reconstruction without manual editing, which is not feasible for large-scale data. For example, the R package PhyTools (Revell 2012) has not implemented the ability to do reconstruction using data at internal nodes (Liam Revell, personal communication, June 6, 2015), but it has developed a workaround method, which could not be applied at this scale. Thus, an algorithm was developed and applied to propagate data at the genus and family levels to the species contained within these higher level groups, based on the VTO (Fig. 3). Taxonomic levels above family were not considered for propagation. Existing species-level data (asserted or inferred) were never replaced by the propagated species-level data. Species in the VTO previously lacking data were thus automatically added to the data matrix with the propagated data. During the propagation process, all higher-level taxa were removed from the matrix, and the propagated matrix (Supplementary Material Matrix S3 available on Dryad) contained data only at the species level.

#### Reconciliation of taxon names.

The synthetic morphological supermatrix from OntoTrace (Supplementary Material Matrix S1 available on Dryad) contained taxon names from the (VTO; Midford et al. 2013). These names needed to be reconciled with those in the Teleostei species-level tree from Open Tree before merging the data with the tree. This was achieved by first matching taxon names using NCBI Taxonomy IDs (Sayers et al. 2009) as the common identifier, then matching the remaining taxa using exact taxon name matching. This step generates the final output matrix of the pipeline (Supplementary Material Matrix S4 available on Dryad; Fig. 3), which can then be merged with the tree file using phylogenetic software.

### Merging Final Output Matrix with Teleostei Species-level Tree

Once names were reconciled between the Teleostei species-level tree (Supplementary Material File S1 available on Dryad) and the final output matrix (Supplementary Material Matrix S4 available on Dryad), they were merged into a single NEXUS file (see merged tree matrix; Supplementary Material File S3 available on Dryad) using Mesquite v3.10 (Maddison and Maddison 2016). With the tree file open in Mesquite, the final output matrix was added by merging incoming names with the taxon names in the tree (Fig. 3). Terminal taxa with no associated pectoral fin or pelvic fin data remained coded as unknown (“?”) in the final resulting merged tree matrix (Supplementary Material File S3 available on Dryad).

### Ancestral State Reconstruction

Ancestral state reconstruction (Fig. 3) was performed with unordered Fitch parsimony, with the cost of changing from one state to another counted as one step, to calculate the total number of steps corresponding to the instances of gain and loss across teleosts. This model was used instead of likelihood methods, as the tree lacked branch lengths and because polymorphic species are not currently supported by categorical data likelihood calculations in Mesquite. Although branch lengths could potentially be estimated for more informative distance-based optimization methods, but with such a large highly unresolved phylogeny, it would not likely provide additional information. However, ancestral reconstruction with a standard parsimony method provided insight into where major transitions of interest occur along the branches. Mesquite was used to summarize state changes over trees to determine the minimum and maximum number of gains and losses across all Most Parsimonious Reconstruction (MPR) mappings. To determine the minimum number of regains of a trait following loss, polytomies were randomly resolved and branch lengths were computed using the R package APE (Paradis et al. 2004) and summarized using Mesquite. Tree visualizations were created using the Interactive Tree Of Life (iTOL: Letunic and Bork 2007).

## Results

### Synthetic Morphological Supermatrix

The synthetic morphological supermatrix from OntoTrace (Supplementary Material Matrix S1 available on Dryad) was comprised of two characters (pectoral fin and pelvic fin) associated with 3047 taxa (2663 species, 132 genera, 223 families, and 29 supra-familial taxa) from 87 studies (Supplementary Table S1 available on Dryad). Higher-level taxa (genus, family, and order) were included as taxonomic units in 30 of the 87 studies. Of the 4853 populated cells (of 6094 total) in the synthetic morphological supermatrix, 616 contained only directly asserted data, 3953 contained only inferred data, and 284 contained both asserted and inferred data. For pectoral fin, 246 taxa have only asserted data, 2020 taxa have only inferred data, and 42 taxa have both asserted and inferred data. For the pelvic fin, 370 taxa have only asserted data, 1933 taxa have only inferred data, and 242 taxa have both asserted and inferred data.

Apparent polymorphic character states and conflicts were identified from 74 taxa (50 families and 24 genera for pelvic fin and 4 families for pectoral fin) and removed from the matrix. Actual polymorphism, that is, within species variation identified by a single author, was found for only the pelvic fin (in five species: a catfish, *Glanapteryx anguilla*, Nelson 2006; two hatchet herrings, *Pristigaster cayana* and *Pristigaster* sp. Di Dario 1999; and two priapumfishes, *Phallostethus lehi* and *Phallostethus dunkeri*, Nelson 2006). Conflicts at the species level that were automatically generated in the process of data aggregation and inference were all between asserted and inferred states. These were found in the pelvic fin for five species (the eel catfish, *Channallabes apus*, two air-breathing catfishes, *Dolichallabes microphthalmus,* and *Gymnallabes typus*, the cobia, *Rachycentron canadum*, and the three-spined stickleback *Gasterosteus aculeatus*), and in the pectoral fin for one species (the bobtail snipe eel, *Neocyema erythrosoma*). Conflicts at the species level, as well as the species polymorphisms, were retained in the matrix because they did not influence the propagation step.

### Teleostei Species-level Tree

The Teleostei species-level tree retrieved from Open Tree (Supplementary Material File S1 available on Dryad) contained 38,419 species-level tips and 560 families (https://tree.OpenTreeoflife.org/about/taxonomy-version/ott2.10). The reference taxonomy used by Open Tree for taxonomic data for fishes is based on the National Center for Biotechnology Information (NCBI), the Interim Register for Marine and Non-marine Genera (IRMNG), the Global Biodiversity Information Facility (GBIF), and the World Register of Marine Species (WoRMS); none of these sources includes fossil species labeled as such. Further, the Open Tree taxonomy does not contain the single expert source of valid species names for fishes, the Catalog of Fishes (Eschmeyer et al. 2013), which is the backbone for the VTO. Because species lists from CoF are not made publically available for download, we used Fishbase, which ingests CoF, as a proxy. The teleost Open Tree shares 30,258 species with Fishbase and includes 8161 unique species (Supplementary Table S2 available on Dryad). Of these unique species, 5958 are not of the form “Genus species” (e.g., those with BOLD identifiers, such as Acanthemblemaria sp. BOLD: AAB1274). Others appear to be invalid names (e.g., *Abramis parsa*). Also, because taxonomic inclusion varies among sources, it is not surprising that the number of families contained in Open Tree differs from those in the CoF and the VTO (families in OT: 560, CoF: 488, VTO: 526; Supplementary Table S3 available on Dryad).

### Data Propagation

Propagation using the relationships in the VTO taxonomy hierarchy transferred asserted and inferred data from 182 families and 119 genera to the member species that otherwise lacked data. This resulted in the addition of 11,293 species to the pre-processed data matrix of 2663 species (Supplementary Material Matrix S2 available on Dryad) for a total of 13,956 species in the propagated matrix (Supplementary Material Matrix S3 available on Dryad). A comparison of propagated data with directly asserted and inferred data revealed ten instances of conflicts with asserted and two with inferred data.

### Reconciliation of Taxon Names

Using the combined method of name reconciliation, first with NCBI taxonomic IDs and then exact taxon name matching, an efficient method of alignment between the 13,956 species associated with the propagated matrix (Supplementary Material Matrix S3 available on Dryad) and 38,419 species in the Open Tree was achieved: 12,582 of the 13,956 species were matched with tree tips. This is higher than using either method alone (NCBI taxonomic IDs: 4423 matches; exact taxon name matching: 12,500 matches). Of the unmatched species (1374 of 13,956), 72 are fossil species which are not included in the Open Tree taxonomic sources, 362 are species with unconventional names that were added to the VTO because they are referenced in publications (e.g., “*Notropis sp. sawfin shiner* (Coburn and Cavender 1992)”), and 940 are unmatched for multiple reasons (e.g., taxonomic name changes, extinct species that are not marked as such in the VTO; Supplementary Table S4 available on Dryad).

Before propagation, the pre-processed data matrix (Supplementary Material Matrix S2 available on Dryad) contained only 2663 species for two characters (pectoral fin and pelvic fin), with 3538 populated cells for species (85.9% missing data; Table 1). The final output matrix (Supplementary Material Matrix S4 available on Dryad) contained 12,582 species with 16,408 populated cells (34.8% missing data; Table 1). When the final output matrix was merged with the Teleostei species-level tree, however, the missing percentage increased to 78.7% in relation to 76,838 total cells in the merged tree matrix (Supplementary Material File S3 available on Dryad; Table 1). Hypothetically, if the pre-processed matrix were merged with the Teleostei species-level tree before propagation, the percentage of missing data in this matrix would be considerably higher (95.4%; Table 1).

**Table 1.** Percentage of missing data before and after data propagation

	Cells with data for pectoral fin	Cells with data for pelvic fin	Total populated cells	Percentage of missing data in the final output matrix	Percentage of missing data in the merged tree matrix
Before propagation (pre-processed matrix)	1661	1877	3538	85.9%	95.4%
After propagation (final output matrix)	10,459	5949	16,408	34.8%	78.7%

*Notes:* The change in the percentage of missing data before propagation in the pre-processed matrix (Supplementary Material Matrix S2 available on Dryad) compared to after propagation in the final output matrix (Supplementary Material Matrix S4 available on Dryad). Missing percentages relative to the total number of species in the final output matrix (12,582 species; 25,164 cells) versus those in the merged tree matrix (38,419 species; 76,838 cells; Supplementary Material File S2 available on Dryad).

**Figure 4.**
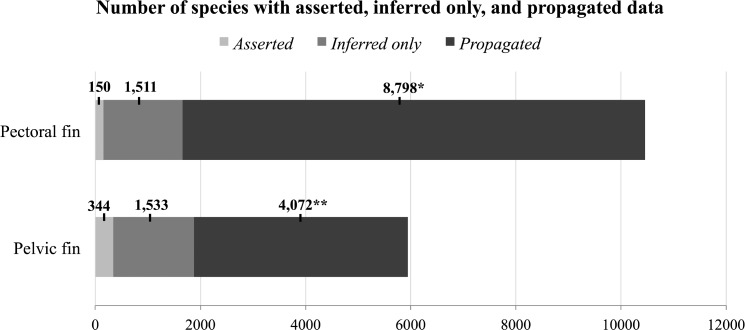
Combined usage of inference and propagation extends morphological data. The bar chart shows the number of species with asserted (light gray), inferred only (dark gray), and propagated (black) data for the pectoral fin and pelvic fin. Increase in the number of species with data after inference and then propagation demonstrate the importance of these steps in reducing missing data. *Of the 8798 species for which pectoral fin data are propagated from family and genus-level data, 5077 are propagated from asserted data, and 3721 are propagated from inferred data. **Of the 4072 species for which pelvic fin data are propagated from family and genus-level data, 2906 are propagated from asserted data, and 1166 are propagated from inferred data.

Of the 16,408 populated cells in the final output matrix, 494 (150 pectoral, 344 pelvic) contained only directly asserted data (Fig. 4). The presence of the pectoral fin was asserted in 123 species, and absence asserted in 30. The presence of the pelvic fin is directly asserted in 150 species and absence asserted in 194. In the remaining cells, 3044 (1511 pectoral, 1533 pelvic) contained only inferred data, and 12,870 cells (8798 pectoral, 4072 pelvic) contained propagated data (Fig. 4). Of the 8798 species for which pectoral fin data are propagated, 5077 of these are propagated from asserted family and genus-level data. Of the 4072 species for which pelvic fin data are propagated, 2906 of these are propagated from asserted family and genus-level data.

### Ancestral State Reconstruction

**Figure 5.**
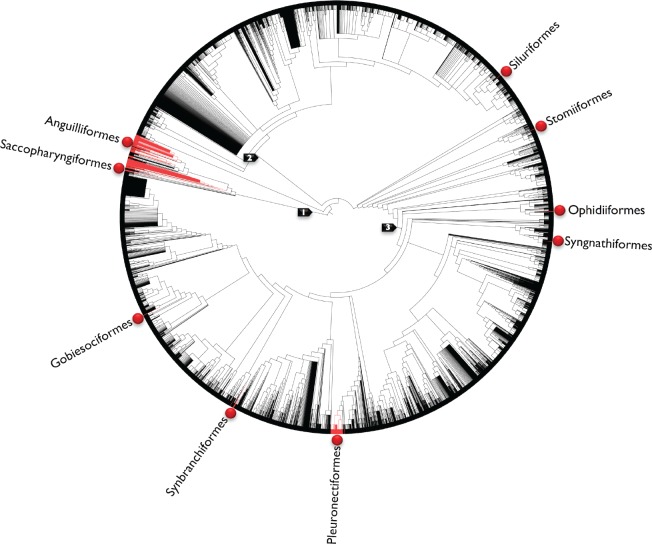
Visualization of pectoral fin presence (black) and absence (red) across 38,419 species of teleost fishes using an unordered parsimony method of reconstruction requiring 27 steps. Fin loss is evident in nine orders (red balls). Arrows indicate higher-level groupings: 1 $=$ Elopomorpha; 2 $=$ Otomorpha; 3 $=$ Percomorphaceae.

**Figure 6.**
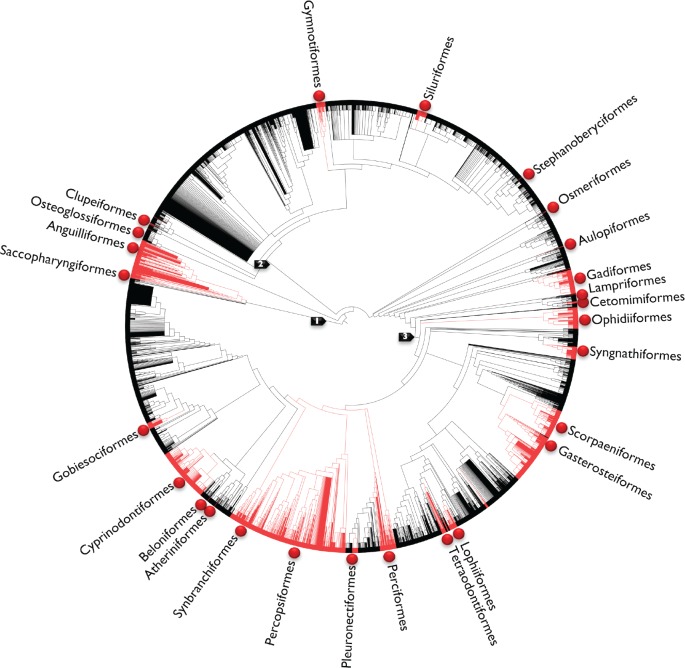
Visualization of pelvic fin presence (black) and absence (red) across 38,419 species of teleost fishes using an unordered parsimony method of reconstruction requiring 80 steps. Fin loss is evident in 26 orders (red balls). Arrows indicate higher-level groupings: 1 $=$ Elopomorpha; 2 $=$ Otomorpha; 3 $=$ Percomorphaceae.

The pectoral fin is absent in 509 of the 12,582 matched species in the final output matrix (Supplementary Material Matrix S4 available on Dryad), and 21 of the 526 teleost VTO families (Supplementary Table S5 available on Dryad) across nine different orders (Fig. 5). Of the 21 families, 17 families (494 species) also have pelvic fin absence, with four families (15 species) lacking only the pectoral fin (Fig. 4). Two of the 21 families with pectoral fin absence involve ontogenetic loss (the swamp eels, Synbranchidae, Nelson 2006; William’s tonguefish, *Symphurus williamsi*: Cynoglossidae, Aceves-Medina et al. 1999). The pelvic fin is absent in 2140 of the 12,582 matched species, and 108 of the 526 teleost VTO families (Supplementary Table S5 available on Dryad) across twenty-six different orders (Fig. 6). This does not include Siluridae and Schilbidae, where family-level assertions of absence were untraceable to species. Ninety-two (1652 species) of the 108 families lack only a pelvic fin; 17 families also lack the pectoral fin.

Based on the Open Tree phylogeny, in which pectoral fin presence is the ancestral condition for Teleostei, there were 73,728 MPRs for pectoral fin evolution, each requiring 27 steps. A summary over 1000 randomly sampled MPRs show a minimum of 19 losses and a minimum of 3 regains of the pectoral fin (Table 2). All regains occurred in the eels (Anguilliformes), and losses occurred in Anguilliformes, Gobiesociformes, Ophidiiformes, Pleuronectiformes, Saccopharyngiformes, Siluriformes, Stomiiformes, Synbranchiformes, and Syngnathiformes (Fig. 5). For the pelvic fin, in which presence is also the ancestral condition for Teleostei, there were 99,777,458,995,200 MPRs, each requiring 80 steps. A summary over 1000 randomly sampled MPRs required the regain of the pelvic fin following a loss a minimum of 14 times, and with a minimum of 48 loss events (Table 3). For the pelvic fin, this occurred primarily within Perciformes, but also within 25 additional orders, such as Anguilliformes, Lophiiformes, Ophidiiformes, and Synbranchiformes (Fig. 6). The greater number of MPRs for the pelvic fin is likely because of the larger number of tips (2140) with absence data compared with the pectoral fin (509).

**Table 2.** Pectoral fin: summary of state changes for gain and loss

Change	Minimum steps	Maximum steps	Average steps over all MPRs
0 $\to $ 1 (gain)	3	7	4.9
1 $\to $ 0 (loss)	19	23	21.5

*Notes:* Summary of state changes over the Teleostei species-level tree for gain and loss of each trait using a Fitch unordered parsimony method with equally weighted transitions. Minimum, maximum, and average number across all mappings and trees for pectoral fin reconstruction requiring 27 steps.

**Table 3.** Pelvic fin: summary of state changes for gain and loss

Change	Minimum steps	Maximum steps	Average steps over all MPRs
0 $\to $ 1 (gain)	14	27	19.1
1 $\to $ 0 (loss)	48	64	56.1

*Notes:* Summary of state changes over the Teleostei species-level tree for gain and loss of each trait using a Fitch unordered parsimony method with equally weighted transitions. Minimum, maximum, and average number across all mappings and trees for pelvic fin reconstruction requiring 80 steps.

**Figure 7.**
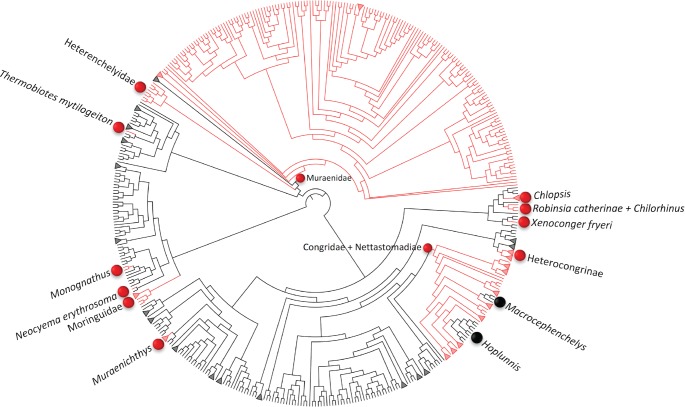
Visualization of pectoral fin presence (black) and absence (red) across the 1073 species of eels (Anguilliformes). The fully resolved phylogeny shown here is one of the 1000 randomly resolved topologies requiring the minimum number (2) of pectoral fin regain events. Pectoral fin presence is the ancestral state for Anguilliformes; red balls represent fin loss. Black balls show the taxa in which regain of the pectoral fin has occurred. Collapsed branches represent subfamilies or genera having five or more species sharing the same character state.

Because ancestral reconstruction across all teleost fishes suggested that the pectoral fin has been regained a minimum of three times in Anguilliformes, we investigated this in more detail. A comparison in the reconstruction was done using various topologies with the following parameters: unordered parsimony, computing branch lengths and performing Mk1 likelihood, randomly resolving polytomies, and assuming missing data as presence. This showed that random resolution of 1000 polytomies (Supplementary Material File S4 available on Dryad) in Anguilliformes minimized the number of regains to two (Fig. 7; Supplementary Table S6 available on Dryad).

## Discussion

Addressing many questions in organismal and evolutionary biology requires knowledge of the traits that species possess or lack, the evolutionary relationships of those species, and the integration of this knowledge—that is, a mapping of the traits to trees. Many excellent examples have demonstrated the value of this approach, primarily by acquiring the trait data through direct observation of a limited number of species and mapping it to a companion phylogeny that is often generated using molecular data. The availability of trait data, however, is limited by the time required for traditional data acquisition, whereas large phylogenies are increasingly available because of the ease of collection and analysis of molecular data. Concatenation of trait data across different characters and taxa that are reported in dispersed studies have been manual, and thus rarely accomplished on a large scale. As a result, trait data are sparse, even for relatively simple traits, such as presence/absence of a structure.

It was recently shown that morphological data can be readily integrated across matrices by annotating it with ontologies and thus making it computable (Ramírez et al. 2007; Dahdul et al. 2010; Walls et al. 2012; Deans et al. 2015). A substantial level of missing data, however, is the inevitable result of combining separately published morphological data matrices each containing different sets of characters and taxa. Ontology annotations, however, enable the extension of sparse morphological data to additional species through inference (Dececchi et al. 2015).

Uniquely in this study, we used phylogenetic propagation, that is, transferring data from families and genera to included species, to further extend the data. In doing so we increased the number of species with data and dramatically reduced missing data to 34.8% in the final output matrix (Supplementary Material Matrix S4 available on Dryad; Fig. 4). The use of propagation was motivated by the goal to preserve the data associated with higher-level taxa, which were included as taxonomic units in over a quarter of the phylogenetic studies mined in the assembly of the supermatrix. There were several considerations in this process. First, we propagated the data from genera and families, but not higher-level ranks, given the increasing expectation of evolutionary changes in character state with increasing divergence time. Second, we eliminated all annotations of polymorphisms (“0&1”) to higher-level taxa so that they were not propagated. Authors use these annotations, which we termed “apparent polymorphisms,” as a shorthand to indicate that both the presence and absence of a trait are found in the species contained in the higher-level clade. In these cases the identity of the specific species that possess or lack a particular structure is not provided. Propagating both states to descendant species would be uninformative and misleading, and hence we removed these annotations. Third, we never propagated data from higher-level nodes to species that had existing data. That is, if a species had either asserted or inferred data, it was never “overwritten” by data propagated from the higher-level node. However, we found that the risk of propagating erroneous data to species is in fact relatively low, at least for fin presence/absence: only 12 of the 11,293 species would have had erroneous data propagated to them. Fourth, we used care at the time of annotation to match the author’s intended higher-level taxon with the corresponding member species in the taxonomy used in constructing the supermatrix. However, we recognize the risk of propagating the data to species unintended by the original author because of changes in taxonomic inclusion (Franz et al. 2015). For example, Callichthyidae, according to Nelson (2006), possess pectoral fins and consist of about 8 genera and 177 species. However, Callichthyidae in the VTO (derived from CoF; Midford et al. 2013), consist of 9 genera and 211 species. Because “pectoral fin present” was annotated to “Callichthyidae” as per the VTO, this trait was linked to each of these 211 species following propagation. These data are now applied to species not considered by the original author, thus clearly incurring a risk that they may be incorrect.

After propagation, nearly twice as many species had data for the pectoral fin versus the pelvic fin (Fig. 4), though before propagation, the number is similar between fins. This is because the number of families and genera from which data are propagated is higher for the pectoral fin (151 families, 97 genera) versus pelvic fin (90 families, 71 genera). In addition, most of the families in the VTO to which pectoral fin data are annotated are more speciose (e.g., Loricariidae, 899 species; Callichthyidae, 211 species) than those for which there are pelvic fin data (e.g., Congridae: 214 species; Synodontidae: 71 species).

The question of confidence arises with respect to data that are generated through inference. Clearly a high level of certainty can be associated with direct author statements concerning morphological features at the species level, particularly if they are associated with voucher specimens. For example, the asserted absence of the pelvic fin and girdle in the knifefish (*Apteronotus apurensis*, Albert 2001) comes from the author’s observations on voucher specimens that are housed in various collections, for example, the Field Museum of Natural History (FMNH). The specimen lot numbers provide access to the specific individuals on which the observations were made (e.g., FMNH 85499, FMNH 100738). A similar level of confidence can be associated with the inferred presence or absence of morphological features, particularly if inferred from an observation on vouchered material. For instance, the presence of a pelvic fin in the armored catfish (*Acanthicus hystrix*) was inferred based on the observation by Armbruster (2004) on vouchered specimens that “two rows of the first pelvic-fin ray are fused” in this species. Even though the author did not directly state that the pelvic fin is present, the logic leading to an inference of presence is based on their reproducible observation.

Though large-scale molecular phylogenies are increasingly obtainable, they are rarely available at the scale required by this study (33,000$+$ tips). Supertree approaches are required to synthesize previously published phylogenetic trees. The Open Tree supertree pipeline yielded a phylogeny that to our knowledge is the most comprehensive synthetic tree ever assembled for teleosts, harvesting 200 source phylogenies (Supplementary Material File S2 available on Dryad). In contrast, a recent large supertree assembled for a study on basal vertebrates relied on 118 source trees (38 for teleosts) and had only 2730 tips across extinct and extant fishes (Larouche et al. 2017). The Open Tree approach affords access to all source trees, node-based provenance, and brings in species that may not have been included in source trees through a reference taxonomy. The latter point was critical in this work, because many of the species with trait data had not previously been studied phylogenetically, thus not available in source trees.

### Opportunities, Challenges, and Future Directions in Integrating Traits and Trees

Two developments made the opportunity ripe for this study: huge phylogenies with provenance, and computable traits with tools for aggregation into a supermatrix. Taxonomic reconciliation, that is, matching taxa from different sources, was the primary challenge involved in merging the trait data from the KB with the phylogenies provided by Open Tree. Neither Phenoscape nor Open Tree is attempting to develop a taxonomic standard; both are harvesting a subset of the available resources. One source of differences in the case of teleost fishes is the lack of incorporation of the expert fish reference taxonomy (CoF) into the Open Tree taxonomy. The impact of the different taxonomic composition of these sources was marked: valid species with data were lost, and species with no data, mainly all of which do not have valid names, were added. Over 8000 species in the teleost Open Tree are not recognized in the Catalog of Fishes (Supplementary Table S2 available on Dryad) and thus lacked data on the phylogeny. These tips contribute substantial uncertainty and noise to the optimization results. Future unification of disparate sources by the broader taxonomic community will reduce some reconciliation issues.

Taxonomic reconciliation across multiple data sources is an active research area, and current methods frequently use the taxon name as the integrative unit (Patterson 2003). This introduces several challenges, such as resolving synonyms, abbreviations, misspellings, and handling improper naming syntax (Cranston et al. 2014; Patterson et al. 2016). Moreover, homonyms can exist when a single taxonomic name belongs to multiple tips within the same taxonomy (Rees and Cranston 2017). Available solutions include using online servers that perform name resolution, such as Taxonomic Name Resolution Service (TNRS), which act as scientific name repositories that aggregate data from different sources (Boyle et al. 2013), and the use of software like the toolkit distributed by Global Names Architecture (GNA; Patterson et al. 2016). The Open Tree integrates multiple source taxonomies to build the Open Tree taxonomy, and names are matched from individual taxonomic sources to identify the correct taxonomic name (Redelings and Holder 2017; Rees and Cranston 2017). However, these solutions do not support the VTO, which thus required us to develop another method for efficient reconciliation. Taxonomy ID can be used as an alternative for taxon name (Thomson and Shaffer 2010). However, depending solely on NCBI taxonomy IDs for reconciliation was inefficient because a large number of VTO taxa (9522) in the propagated matrix did not have any reference to NCBI taxonomy IDs. Therefore, two reconciliation methods—based on taxon name and NCBI taxonomy IDs—were integrated in this work. Taxonomic reconciliation at large scale, however, remains a major challenge.

A second major technical challenge in this work was the lack of tools that support visualization and manipulation of trees at this large scale. Branch navigation on large-scale phylogenies is cumbersome, and manual efforts to investigate state changes along branches are difficult. The next generation of tools must facilitate navigation to specific nodes and support more complex analyses (Gruenstaeudl 2016).

A third challenge involves the lack of branch lengths in the synthetic tree, which limits the analysis of character evolution. Clearly the transitions between finned and finless states are not symmetric. If branch lengths could be considered, it would allow more robust distance-based analyses to be performed, which can account for models of asymmetric change. A putatively high cost transition (e.g., regain of fins after loss), for example, would be more likely on a long branch than a short one. This would enable an understanding of the parameters under which fin regain is likely and where additional phylogenetic analysis might valuable in clarifying highly unlikely transitions. As recently observed (Pyron 2017), little attention has been paid to the dynamics of evolutionary transitions in discrete morphological characters; the addition of branch lengths to synthetic trees would enable the use of additional models.

Additional challenges included the substantial level of curation required to develop and maintain resources for phylogenetic trees and ontology-based trait data. Manual curation of trait data is manual and time-consuming (Dahdul et al. 2015), as is the curation of phylogenies for Open Tree. The addition of new natural language processing (NLP) machine-learning curation methods is critical. The approach here clearly demonstrates the value of integrating these data, and it highlights the need for automated tools.

Finally, the methods and pipeline demonstrated here to integrate a large-scale morphological supermatrix from the Phenoscape KB with a Teleostei species-level tree from the Open Tree of Life are currently limited in generalizability. Currently the pipeline functions for a matrix that includes only the pectoral and pelvic fins, though the methods and algorithms in the code can be adapted for other characters. A future goal of this work is to develop a more generic pipeline to integrate any large-scale morphological data set coming from the Phenoscape KB with any large-scale phylogeny.

### Data Conflicts Motivate Future Studies

One of the benefits of machine reasoning, as previously pointed out (Dececchi et al. 2015), is that conflicts in the data are automatically isolated. That is, cells where both presence and absence are indicated for a particular trait in a single taxon are made obvious and data provenance is available to further investigate the conflict. Conflicts may result from differing assertions among authors, which may in turn be due to observations of different specimens or different interpretations of the same material. There may also be conflicts between asserted and inferred data, and these were the most common type reported by Dececchi et al. (2015). In the supermatrix generated here, only 0.04% of the species-level data (6 of 16,408 populated cells) were conflicted, excluding actual polymorphisms. All six conflicts were between asserted and inferred data. For example, the presence of the pelvic fin in the airbreathing catfish *Gymnallabes typus* was inferred from a character describing the thickness of its first pelvic fin ray (De Pinna 1993). However, a different author asserted that the pelvic fin was absent for this species (Nelson 2006). In another example, Poulsen (2015) observed “transparent pectoral fin lobes” in the bobtail snipe eel (*Neocyema erythrosoma*), but noted the apparent absence of the pectoral skeleton. The annotation “pectoral girdle skeleton, absent,” however, from which the absence of a pectoral fin is inferred (Fig. 2), results in a conflict with the same author’s assertion of pectoral fin presence. The inference reflects the anatomical knowledge formalized in the ontology, that the presence of a pectoral fin is dependent on the presence of a pectoral girdle (Fig. 1). In fact, the presence of the fin without the underlying girdle has not been reported in any other species. This conflict, like all of the conflicts surfaced through such automated syntheses, leads back to an examination of the evidence. Here we discovered that only five specimens of this rare species have ever been collected (DeVaney et al. 2009; Poulsen 2015), and absence of the pectoral skeleton was described as “apparent” (Poulsen 2015); this raises the possibility that the pectoral girdle may in fact be present. On the other hand, *Neocyema* is a member of a group of deep-sea fishes (Saccopharyngiformes) with many reduced skeletal features, and perhaps the loss of the pectoral girdle is another instance of reduction. This particular conflict, like the others, identifies species and features for further investigation.

### How Often Were the Paired Fins Lost (and Regained)?

The questions of how often, and in which taxa, paired fins were lost have been the subject of many studies in ichthyology, and our work not only demonstrates how these can be answered automatically and at scale, but it also provides species-level data from disparate sources in a phylogenetic framework to provide insight into fin evolution at the macroevolutionary scale. Previous investigators have concluded that pelvic fins appear to be more readily lost or reduced across teleosts than other fins (Nelson 1990; Yamanoue et al. 2008; Larouche et al. 2017) and possibly with greater frequency than other structures, including for example, scales (Nelson 1990) and the gas bladder (McCune and Carlson 2004). The expectation that pectoral fin loss is much rarer than pelvic fin loss (Yamanoue et al. 2010) was born out by our analysis, which indicates more than double the minimum number of independent losses of the pelvic fin (48) compared to the pectoral fin (19) for the phylogeny under consideration (Tables 2 and 3).

The apparent ease of pelvic fin loss has been examined from a genetic standpoint in several species, including the well-known example of the three-spined stickleback (*Gasterosteus aculeatus*; Chan et al. 2010), the Japanese pufferfish (*Takifugu rubripes*; Tanaka et al. 2005), and the tiger tail seahorse (*Hippocampus comes*; Lin et al. 2005). These studies showed different genes or enhancers responsible for pelvic fin loss, indicating that similar phenotypes can evolve by different mechanisms. Studies focused on potential genetic differences that may underlie the difference in frequency of pectoral versus pelvic fin loss, however, have not yet been done.

With respect to pectoral and pelvic fin evolution, there are contradictory expectations as to whether reversal following loss has (Yamanoue et al. 2010) or has not (Nelson 1990) occurred. There is a large literature on the irreversibility of evolution and Dollo’s law (Gould 1970; Farris 1977; Wagner 1982; Kohlsdorf and Wagner 2006; Klimov and OConnor 2013), yet also examples of putative reversals, for example, digit regain within multiple lineages of squamates (Kohlsdorf and Wagner 2006). Genetic studies suggest that the persistence of developmental pathways may provide a route for reversal (Collin and Miglietta 2008).

Our analyses indicate that a substantial number of reversals have occurred in the evolution of the paired fins (minimum of 2 for pectoral fins; 14 for pelvic fins), though interpretation is compromised by the species-level polytomies throughout the teleost tree and the lack of branch lengths, which limit the optimization methods that can be used. To determine whether polytomies could be resolved such that reversal was not required, we focused on one monophyletic clade, the eels (Anguilliformes), in which most of the losses and all of the putative regains of the pectoral fin occurred. This was a clade, in fact, where we manually curated recent phylogenies (Santini et al. 2013; Tang and Fielitz 2013) into Open Tree, and the phylogeny (Fig. 5) is up-to-date and resolved with respect to the most recently published studies for Anguilliformes. Our analysis showed that among 1000 different randomly resolved topologies, the pectoral fins re-evolved following loss a minimum of two times: once within *Hoplunnis* (duckbill eels with nine species; Family: Nettastomatidae) and again in *Macrocephenchelys* (conger eels with two species; Family: Congridae; Fig. 7). This points to species where regain is a very strongly supported hypothesis that could be examined further using genetic tools, for example. Additionally, it points to areas in the tree for strategic resolution, that is, regions of the tree where evolutionary questions, such as understanding the phylogenetic pattern and frequency of reversals and their biological basis, could be answered through further phylogenetic analysis.

## Conclusions

The opportunity to understand the broad patterns of evolution of organismal features, foundational knowledge for many types of studies, is at hand with the tools in place to reuse and synthesize the data. The time-consuming nature of curating phylogenies and trait data into the formats and databases appropriate for their automatic aggregation into larger-scale synthetic products is an immediate challenge, though cultural shifts with respect to data in the life science community and advances in machine learning are progressing. As our case study shows, rendering traits computable enables the extension of relatively sparse data to taxa for which the presence or absence of a trait had not been directly asserted. Further, by propagating author assertions about features for high-level taxa to species for which asserted or inferred data are missing, we show that trait data can be further and significantly extended. An additional benefit from these automated approaches lies in readily discoverable errors in data or knowledge by uncovering conflicts in the data. Such potential errors are not easily found through manual means.

Given the increasingly broad scope of comparative questions across biology, a sizable and growing legacy literature, and the difficulty and expense of new data collection, automated means of existing data reuse and extension by way of inference and data propagation are critically important. Equally important is ensuring that the users of these products have access to the provenance of the data, including the traits for each taxon and the phylogenetic resolution and the means by which they were generated. This case study also revealed several important technical challenges to such integration. Current difficulties in aligning taxonomic sources impede ready integration, and new tools are needed to analyze and visualize the data on large trees.

Finally, from the standpoint of understanding the relative frequencies of pectoral and pelvic fin loss and regain, this study provides evidence that the pelvic fin is independently lost in more than twice the number of lineages as the pectoral fin, and that these fins were regained several to many times in the course of teleost evolution. The general method outlined here, of an automated mapping and extension of traits mined from dozens of studies, to trees assembled from over a hundred more, offers rapid assessments of trait distribution. These in turn set the stage for in-depth analyses of the potential underlying evolutionary mechanisms.

## Supplementary Material

Data available from the Dryad Digital Repository: http://dx.doi.org/10.5061/dryad.v0s27.
